# Monoclonal gammopathy in prostate carcinoma: a case report and review of literature

**DOI:** 10.1186/s13256-018-1811-z

**Published:** 2018-10-25

**Authors:** Samik Pramanik, Md Jahangir Gazi, Anjan Kumar Das, Nirod Baran Debnath, Salil K Pal

**Affiliations:** 1 Department of General Medicine, Calcutta National Medical College, Kolkata, India; 2 Department of Pathology, Calcutta National Medical College, Kolkata, India; 3 Flat no-1A, 876 Tagore Park, Block-C, Naskarhat, Kolkata, 700039 India

**Keywords:** Monoclonal gammopathy, Serum protein electrophoresis, Prostate carcinoma

## Abstract

**Background:**

Monoclonal gammopathy is commonly associated with plasma cell dyscrasia. However, monoclonal gammopathy without bone marrow plasmacytosis in prostate carcinoma has rarely been reported. The association between the two conditions is not clearly established.

**Case presentation:**

We report a case of metastatic prostate carcinoma in a 65-year-old Indian man with the unusual phenomenon of monoclonal band in gamma globulin region without evidence of bone marrow plasmacytosis.

**Conclusions:**

Monoclonal gammopathy in solid tumor has seldom been reported. This case report highlights the rare association of monoclonal gammopathy with malignant prostatic carcinoma. Therefore, while investigating a case such as this, in an elderly male patient, we should always keep a lookout for any solid tumor foci after excluding multiple myeloma.

## Background

Monoclonal gammopathy is defined as an abnormal increase in each monoclonal protein (M protein or paraprotein) consisting of two heavy chains of the same class and subclass and two light chains of the same type. In contrast, a polyclonal immunoglobulin increase consists of one or more heavy chain classes and both light chain types [1]. M component can be identified as discrete homogenous band on electrophoresis of serum and urine.

Monoclonal immunoglobulin in serum is most commonly found in plasma cell dyscrasias like multiple myeloma and Waldenström macroglobulinemia. Monoclonal band has been detected in a variety of lymphomas and chronic lymphocytic leukemia. Paraproteins have also been reported in some solid tumors, but in low frequency [2].

Here, we report a case of metastatic prostate carcinoma with monoclonal immunoglobulin in serum protein electrophoresis (SPEP) without evidence of bone marrow plasmacytosis.

## Case presentation

A 65-year-old Indian man presented with complaints of chest pain along with fatigue and generalized weakness for 2 months. His chest pain was constant, dull and boring in nature, and all over his chest. He had no history of trauma.

**Fig. 1:**
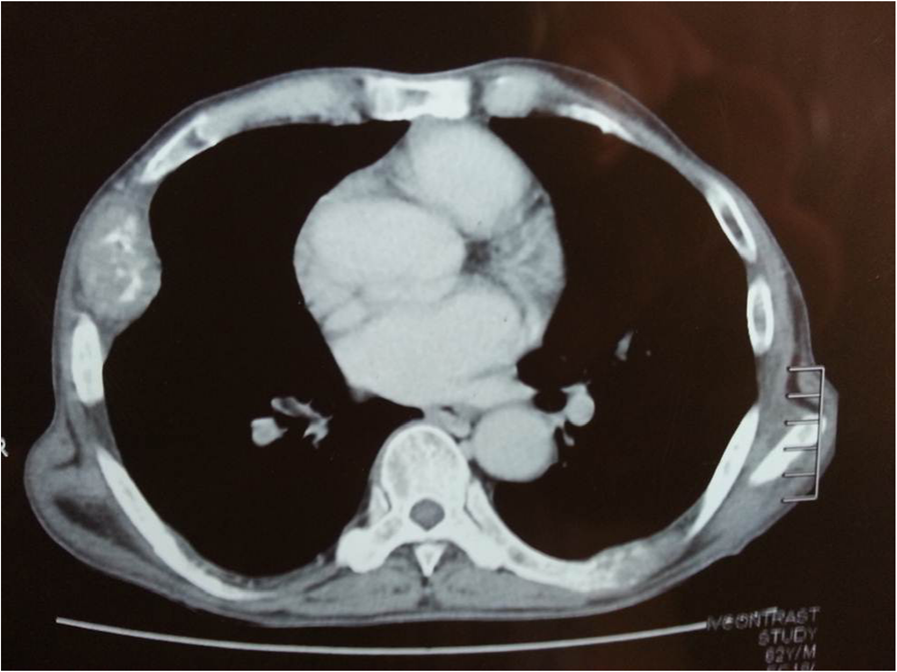
Computed tomography of thorax showed erosion of left 7th & 8th rib and soft tissue mass involving right side of chest wall

Contrast-enhanced computed tomography of his thorax showed erosion of left seventh and eighth rib with soft tissue mass involving the right side of his chest wall (Fig. 1).

**Fig. 2:**
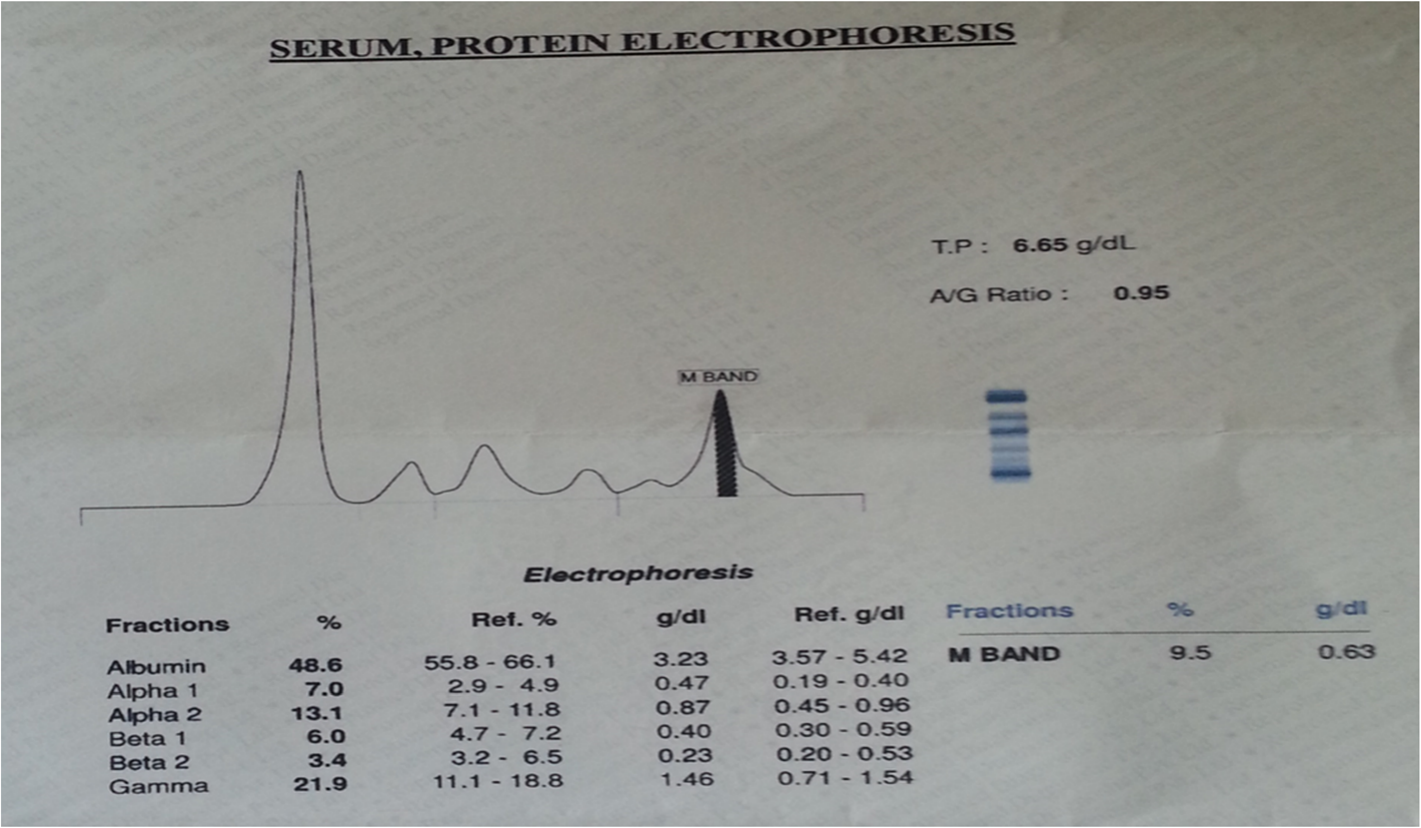
Serum protein electrophoresis demonstrated M band in gamma globulin region

We conducted SPEP of our patient as he had multiple rib erosions. SPEP showed monoclonal band in gamma globulin region (Fig. 2).

**Fig. 3:**
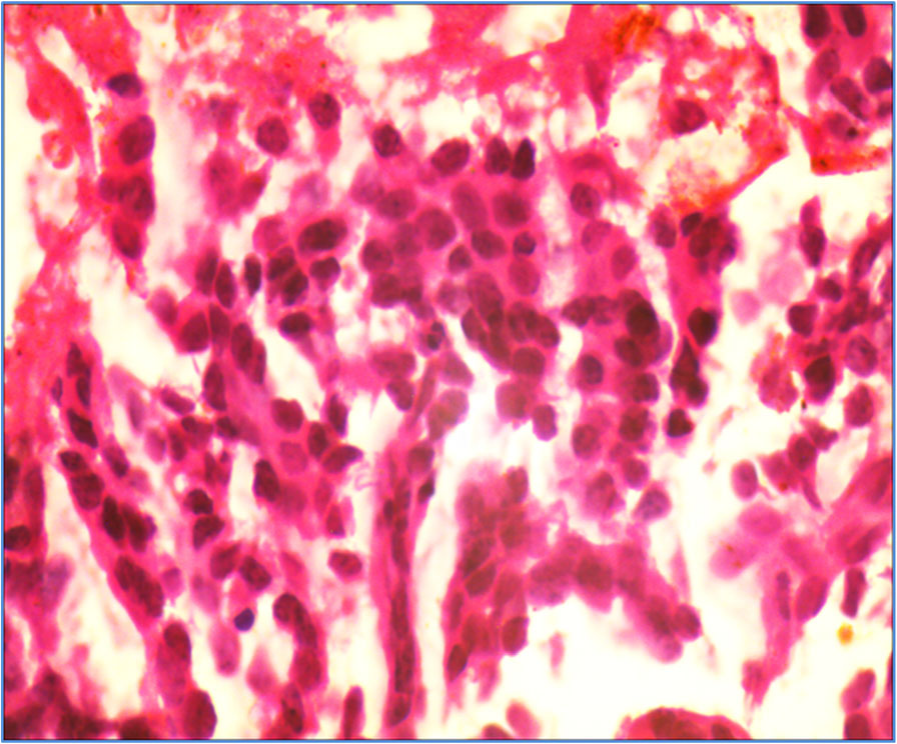
Bone marrow biopsy showed metastatic adenocarcinoma (haematoxylin & eosin, 40X)

Bone marrow aspiration and biopsy were performed to detect multiple myeloma. However, instead, metastatic adenocarcinoma was revealed. No evidence of plasmacytosis was noted (Fig. 3).

**Fig. 4:**
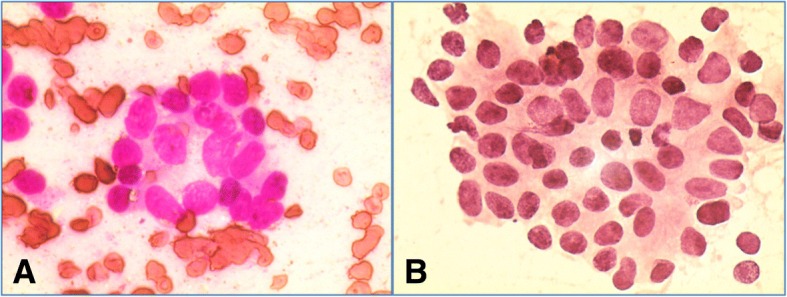
Computed tomography guided fine-needle aspiration cytology (FNAC) from right-sided soft tissue mass showed metastatic adenocarcinoma (A-Leishman & giemsa,40X; B-haematoxylin & eosin, 10X)

**Fig. 5:**
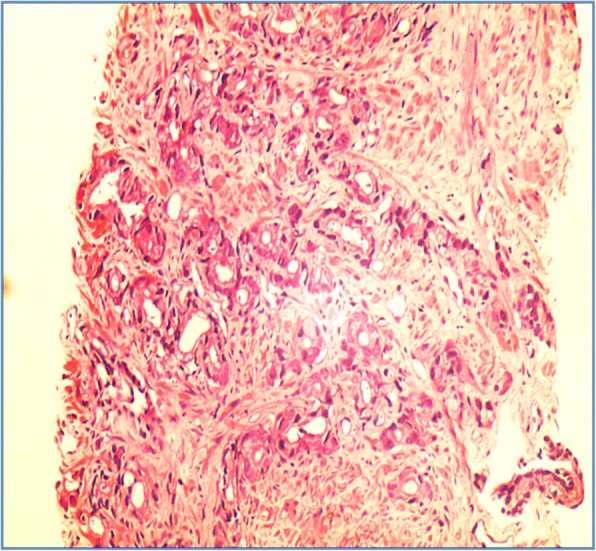
Prostate biopsy showed infiltrative adenocarcinoma with perineural invasion (haematoxylin & eosin,10X)

Computed tomography-guided fine-needle aspiration cytology (FNAC) from right-sided soft tissue mass of his chest wall showed metastatic adenocarcinoma (Fig. 4). On further enquiry, he also gave history of urinary obstruction with lower urinary tract symptoms. Clinical suspicion of prostate carcinoma was considered in our old male patient with metastatic deposit in ribs. A per-rectal examination done by a urologist revealed enlarged hard prostate. Ultrasonography of our patient’s abdomen showed enlarged prostate. Serum prostate-specific antigen (PSA) was 124 ng/ml. A prostate biopsy was performed which demonstrated infiltrative adenocarcinoma with perineural invasion (Gleason’s score 4) (Fig. 5).

A complete hemogram showed hemoglobin 9.4 gm/dl, white blood cell count (WBC) 9900, platelets 280,000, and erythrocyte sedimentation rate (ESR) 89. Blood biochemistry showed Na+ 130, K+ 4.4, Ca+ 1.16 mmol/L, protein 7 gm/dl, albumin 3.4 gm/dl, and globulin 3.6 gm/dl. Liver and renal function tests were within normal limits.

The valuable opinions of the urologist and the oncologist were taken into account. A plan for bilateral orchiectomy was decided upon to control growth of metastatic prostate carcinoma. However, our patient had a sudden cardiac arrest in the preceding week of the planned surgery. Efforts were made to resuscitate him but he died.

## Discussion

Prostate carcinoma remains the second leading cause of cancer deaths in men. Approximately 99% of cases occur in those over the age of 50. Early prostate cancer usually has no symptoms. Metastatic prostate cancer that has to other parts of the body can cause additional symptoms. The most common symptom is bone pain, often in the vertebra, pelvis, or ribs. A monoclonal spike (M spike or paraprotein) on SPEP is a usual finding in a patient with multiple myeloma. The simultaneous association between multiple myeloma and prostate cancer has been described [3, 4]. The possible impact of immunosuppression from multiple myeloma and chemokines released by circulating myeloma cells may lead to progression of prostate cancer [5]. Paraproteins in serum can be detected in a premalignant condition called monoclonal gammopathy of undetermined significance (MGUS) with bone marrow with less than 10% plasma cells [6]. Prostate carcinoma associated with MGUS has been reported [7, 8]. Monoclonal immunoglobulins have often been detected in serum specimens obtained from patients with carcinoma who have no documented evidence of coexisting multiple myeloma [9]. lsobe and Osserman [10], in a review of the clinical and histopathologic data on 806 patients with serum M components noted such proteins in 128 patients with non-reticular neoplasms.

The association between monoclonal band in electrophoresis and cancer is not clearly established. Hellström and Hellström [11] have shown that antibody or complexes of antigen and antibody can block the antitumor effect of cytotoxic lymphocytes, thus facilitating the growth of some clinical and experimental forms of cancer.

## Conclusions

Monoclonal band may be associated with solid tumor. If an elderly man presents with multiple rib erosions with monoclonal band in SPEP, always search for solid tumor after excluding multiple myeloma.

## Abbreviations

ESR: Erythrocyte sedimentation rate
FNAC: Fine-needle aspiration cytology
MGUS: Monoclonal gammopathy of undetermined significance
PSA: Prostate-specific antigen
SPEP: Serum protein electrophoresis
WBC: White blood cell count
